# *In silico* molecular docking and *in vitro* analysis of ethanolic extract *Ocimum sanctum* Linn.: Inhibitory and apoptotic effects against non-small cell lung cancer

**DOI:** 10.14202/vetworld.2021.3175-3187

**Published:** 2021-12-28

**Authors:** Ulayatul Kustiati, T. S. Dewi Ratih, N. Dwi Aris Agung, Dwi Liliek Kusindarta, Hevi Wihadmadyatami

**Affiliations:** 1 Post Graduate Student of Sain Veteriner, Faculty of Veterinary Medicine, Universitas Gadjah Mada, Yogyakarta, 55281, Indonesia; 2 Department of Biology, Faculty of Mathematics and Natural Sciences, and Research center of Smart Molecule of Natural Genetics Resources, Brawijaya University, Indonesia; 3 Department of Pharmacology, Faculty of Medicine, Universitas Gadjah Mada, Yogyakarta, 55281, Indonesia; 4 Department of Anatomy, Faculty of Veterinary Medicine, Universitas Gadjah Mada, Yogyakarta, 55281, Indonesia

**Keywords:** *in vitro*, lung cancer, molecular docking, *Ocimum sanctum*

## Abstract

**Background and Aim:**

Lung cancer, especially non-small cell lung cancer (NSCLC), has been identified as the leading cause of cancer deaths worldwide. The mortality rate from lung cancer has been estimated to be 18.4%. Until now, conventional treatments have not yielded optimal results, thus necessitating an investigation into the use of traditional herbal plants as potential candidates for its treatment. This study aimed to determine the inhibitory and apoptotic activity of the ethanolic extract from *Ocimum sanctum* Linn. (EEOS) by *in silico* molecular docking and through *in vitro* studies using NSCLC cells (A549 cell line).

**Materials and Methods:**

Dried simplicia of *Ocimum sanctum* was converted into EEOS using the maceration method. Spectrophotometry was then employed to analyze the EEOS compound. The known main active compounds were further analyzed for inhibitory and apoptotic effects on gene signaling using *in silico* molecular docking involving the downloading of active compounds from PubChem and target proteins from the Protein Data Bank; the active compounds and proteins were then prepared using the Discovery Studio software v. 19.0.0 and the PyRX 0.8 program, interacted with the HEX 8.0.0 program, and visualized with the Discovery Studio Visualizer v. 19.0. Finally, an *in vitro* analysis was performed using an antiproliferative-cytotoxic test (3-(4,5-dimethylthiazolyl-2)-2,5-diphenyltetrazolium bromide assay in the NSCLC A549 cell line).

**Results:**

The analysis revealed that the active compounds in the ethanolic extract were dominated by quercetin (flavonoids) (47.23% b/b) and eugenol (phenolic) (12.14% b/b). These active compounds interacted with the active sites (residual amino acids) of the αvβ3 integrin, a5b1 integrin, caspase-3, caspase-9, and vascular endothelial growth factor. Hydrogen bonds and Pi-cation and Pi-alkyl interactions were involved in the relationships between the active compounds and the active sites and thus may reveal an antioxidant property of the extract. Furthermore, *in vitro* analysis showed the inhibitory and antiproliferative effects of the EEOS against non-small cell cancer (A549).

**Conclusion:**

Taken together, our data showed the ability of EEOS as an inhibitor and apoptotic agent for lung cancer; however, further research is needed to determine the exact mechanism of EEOS as an herbal medication.

## Introduction

Non-small cell lung cancer (NSCLC), a type of lung cancer, has been determined to be a significant cause of cancer death worldwide. Data from the Global Cancer Statistics show that the mortality rate due to lung cancer was 18.4% in 2018, with 1.8 million deaths and 2.1 million new lung cancer cases recorded [1]. Lung cancer is deemed the most fatal compared with other types of cancer. Although lung cancer is strongly correlated with smoking, adenocarcinoma of NSCLC can also occur in non-smokers, thus increasing the prevalence of lung cancer in all populations.

At present, most lung cancer treatments involve conventional therapy approaches (chemotherapy/medication and surgery). Standard medication therapy in the treatment of NSCLC cases is the use of cisplatin in combination with pemetrexed [2]. In addition, treatment with antibody engineering systems such as pembrolizumab, nivolumab, and atezolizumab is now actively used [3,4]; however, the administration of these drugs has been noted to have drawbacks. Aside from being expensive, these drugs induce a chemoresistant effect that appears in the treatment of lung and breast cancer. In addition, pembrolizumab and nivolumab are known to cause oral mucositis, rush, and pruritus caused by immune-related adverse effects [3,5]. Combination therapy such as radiotherapy with pembrolizumab is known to have side effects that increase the drug’s toxic effect [5].

Because of the disadvantages of these medications, there is a need to explore natural herbal traditional remedies. The system of traditional medicine has been used for thousands of years to prevent, diagnose, and treat several acute and chronic diseases. *Ocimum sanctum* Linn. is a traditional medicine commonly found in Indonesia and Asian countries. Various species of *Ocimum* are known to provide many health benefits, including anti-inflammatory, anti-fatigue, antitussive, antiseptic, antispasmodic, neuroprotective, and neuroproliferative activities [6-11], but until recently, their active mechanisms, safety, and dosage have not been determined.

Thus, in this study, we aimed to determine the dynamic majority content of the ethanolic extract from *O. sanctum* Linn. (EEOS) and analyze the extract’s effect against NSCLC by *in silico* molecular docking and *in vitro* studies.

## Materials and Methods

### Ethical approval

The study was approved by the Ethics Committee of the Faculty of Veterinary Medicine, Universitas Gadjah Mada, Yogyakarta, Indonesia (00053/EC/FKH/Int./2021).

### Study period and location

The study was conducted from January to June 2021 at the Department of Pharmacology Faculty of Medicine, Public Health, and Nursing and Integrated Laboratory for Research and Testing Laboratory, Universitas Gadjah Mada

### Preparation of ethanolic extract

*O. sanctum* Linn. leaves and dried simplicia were derived from the herbal company CV Merapi Farma Herbal, Yogyakarta, Indonesia. The leaves were identified at the Laboratory of Plant Systematics, Faculty of Biology, Universitas Gadjah Mada. The dried simplicia was then made into an ethanolic extract at the Integrated Laboratory for Research and Testing, Universitas Gadjah Mada. The ethanolic extracts were made using the maceration technique. A volume of 4000 mL of ethanol 96% (Merck) was added to the simplicia of *O. sanctum*, which weighed as much as 300.12 g. The mixture was stirred for 30 min, allowed to stand 48 h, and then filtered twice. The filtrate was evaporated using a vacuum rotary evaporator (Buchi, Flawil, Switzerland) and heated in a water bath (Memmert, Schwabach, Germany) at 60°C. The thick extract was poured into a porcelain cup and heated in a water bath at 70°C with occasional stirring. A final extract weight of 26.48 g of *O. sanctum* leaf extract was obtained.

### Spectrophotometry analysis

#### Flavonoid

EEOS, weighing 50 mg, was placed into a 10 mL test tube, in which 0.3 mL of 5% sodium nitrite (Merck) was subsequently added. After 5 min, 0.6 mL of 10% aluminum chloride (Merck) and 2 mL of 1 M sodium hydroxide (Merck) were added to the solution along with the addition of up to 10 mL of distilled water. The mixture was then transferred into a cuvette and measured through spectrophotometry (Shimadzu, Kyoto, Japan) at a wavelength of 510 nm. Quantification of the total compound was calculated using the following formula:


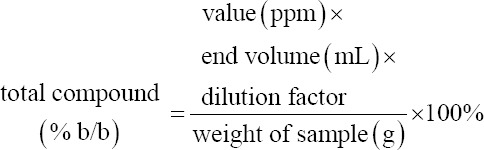


#### Phenol

The EEOS weighed 50 mg. To the extract, 0.5 mL of Folin–Ciocalteu (Merck) reagent and 7.5 mL of aquabides were added. The mixture was allowed to stand for 10 min at 24°C followed by the addition of 1.5 mL of 20% sodium carbonate (Merck). Sterile water was then added to achieve a final volume of 10 mL. The solution mixture was transferred into a cuvette and measured on a spectrophotometer (Shimadzu) at a wavelength of 760 nm. Quantification of the total compound was calculated using the following formula:


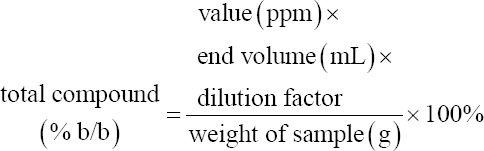


### In silico molecular docking

#### Bioactive compound preparation

The two bioactive compounds with the largest amounts in the EEOS were identified as eugenol (CID_3314) and quercetin (CID_5280343), downloaded from PubChem database (https://pubchem.ncbi.nlm.nih.gov/).

### Protein target preparation

The target proteins used in this *in silico* study were integrin αvβ3 (Protein Data Bank [PDB] ID: 4g1e), integrin α5β1 (PDB ID: 3vi3), caspase-9 (PDB ID:1jxq), caspase-3 (PDB ID: 1nms), and vascular endothelial growth factor (VEGF) (PDB ID: 3v2a), which were downloaded from the Structural Information PDB (https://www.rcsb.org/ligand/).

### Molecular docking analysis

Bioactive compounds (flavonoids and phenols) and target proteins (integrin αvβ3, integrin α5β1, caspase-9, caspase-3, and VEGF) were prepared sequentially using the Discovery Studio v. 19.0.0 program (https://www.3ds.com/products-services/biovia/) and the PyRX 0.8 program. The proteins and bioactive compounds were interacted with the HEX 8.0.0 program (http://hex.loria.fr/) and visualized with the Discovery Studio Visualizer v. 19.0.0 (https://www.3ds.com/products-services/biovia/).

### Maintenance of the NSCLC (A549) cell line

The NSCLC cells (A549 cell line) were generously gifted by Prof. Dr. Srikanth Karnati (Wuerzburg, Germany). The cells were cultured in the complete growth medium, Dulbecco’s Modified Eagle Medium (Gibco, New York, USA) containing 10% fetal calf serum (Capricorn Scientific, Ebsdorfergrund, Germany), penicillin-streptomycin (Capricorn Scientific), and amphotericin (Gibco). The cells were maintained at 37^∘^C in a 5% CO_2_ incubator.

### Cell proliferation assay (3-(4,5-dimethylthiazolyl-2)-2,5-diphenyltetrazolium bromide [MTT] assay)

The inhibitory activity of the EEOS was examined using an MTT assay. Cells that reached an 80% confluence in culture were detached with 1 μL of Accutase cell detachment (Capricorn Scientific). The cells were then inserted into a 15 μL sterile conical tube and centrifuged at 1500 rpm for 5 min. The supernatants were removed, and the cells were counted using a cell counter (Corning, New York, USA). Then, NSCLC (A549) cells were seeded onto a 96-well plate at a density of 1.5×10^4^ cells/well and incubated overnight at 37°C. Afterward, the cells were cultured with the AP3 monoclonal antibody at a concentration of 80 μg/mL as the antiproliferative positive control. In addition, 9 μg/mL cisplatin was run as the positive control of a commercial drug used to treat lung cancer. Furthermore, the EEOS was added in increasing concentrations (50, 70, 100, and 200 μg/mL). The cells were incubated for 24 h, washed with Dulbecco’s phosphate buffer saline (Capricorn Scientific) followed by 10 μL of 5 μg/mL MTT (Merck), and incubated in the CO_2_ incubator (Eppendorf, Hamburg, Germany) for 4 h at 37°C and 5% CO_2_. Finally, the medium was removed, and 100 μL of 95% sodium dodecyl sulfate 10% (Merck) was added to dissolve the formazan crystals. Optical density values were obtained using a microplate reader (Bio-Rad, California, USA) at 595 nm. Inhibition of the A549 cell line samples was calculated using the following formula:


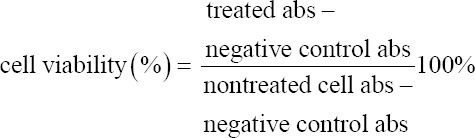


### Observation of cell morphology

The NSCLC (A549) cells were seeded onto 6-well plates (at 5×10^5^ cells/mL) for 24 h and then treated with 80 μg/mL of the AP3 monoclonal antibody inhibitor as the positive control, followed by 9 μg/mL of cisplatin as the commercial drug positive control, and the EEOS at increasing concentrations of 50, 70, 100, and 200 μg/mL for 24 h. The morphology of the cells was observed and photographed using a bright-field microscope (Nikon Eclipse TE2000-E, Tokyo, Japan). The observation data were analyzed semi-quantitatively.

### Statistical analysis

The data obtained were analyzed using a one-way analysis of variance followed by a post hoc test by GraphPad Prism software v. 7 (La Jolla, CA, USA).

## Results

### Eugenol and quercetin are the dominant components in the EEOS

The experimental analysis using an ultraviolet-visible spectrophotometer showed that EEOS consisted mostly of the active compound of flavonoid derivatives, that is, quercetin (47.23% b/b), and phenol derivatives, that is, eugenol (12.14% b/b) (Table-1).

**Table 1:** Bioactive compounds with the largest composition contained in the ethanolic extract of *Ocimum sanctum* Linn.

No.	Name of compound	Concentration	Unit	Method
1.	Quercetin (Flavonoid)	47,23	%b/b	Spectrophotometry UV-Vis
2.	Eugenol (Phenol)	12,14	%b/b	Spectrophotometry UV-Vis

### Quercetin binds to the active sites of integrins α5β3 and α5β1

**Figure-1:**
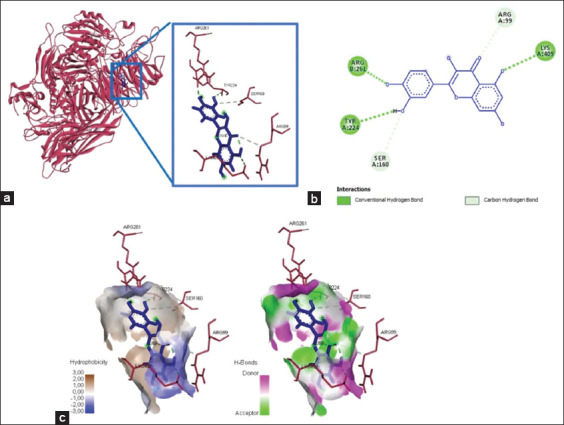
The three-dimensional and two-dimensional structure of the interaction between flavonoid compounds (quercetin) and αvβ3 integrins. (a) Three-dimensional structural interaction between flavonoid-ligand compound quercetin (blue) against αvβ3 integrins on the active binding site (red). (b) This interaction is established by hydrogen and carbon-hydrogen bonds on the active site of the αvβ3 integrin, namely, Arg99, Arg261, Tyr224, and Lys409, which can be seen in the two-dimensional (b) and three-dimensional (c) structures.

**Figure-2:**
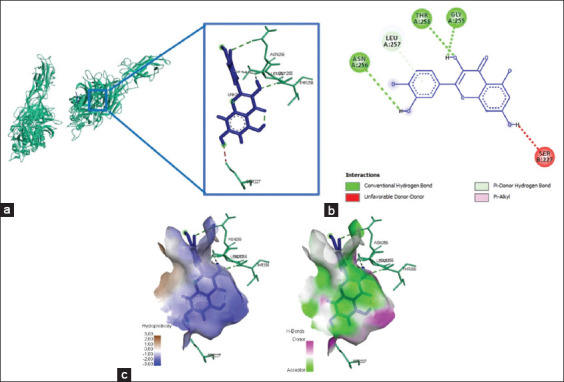
The three-dimensional and two-dimensional structure of the interaction between flavonoid compounds (quercetin) and α5β1 integrins. (a) Three-dimensional structural interaction between flavonoid-ligand compound quercetin (blue) against integrin α5β1 on the active binding site (green). (b) This interaction is built by hydrogen and carbon-hydrogen bonds on the active site of the α5β1 integrin, namely, Thr258, Gly255, Asn256, Leu257, and Ser277, which can be seen in the two-dimensional (b) and three-dimensional (c) structures.

Four amino acid residues depict the binding of quercetin and integrin αvβ3 at Lys409, Arg261, Tyr224, Arg99, and Ser160, yielded bond energy of 257.3 kJ/mol (Figure-1 and Table-2). Meanwhile, the interaction between quercetin and integrin α5β1 showed five interacting amino acid residues (Thr258, Gly255, Asn256, Leu257, and Ser277) and yielded bond energy of −226.1 kJ/mol (Figure-2 and Table-2). The bonding between quercetin and αvβ3 and α5β1 occurred through hydrogen and carbon-hydrogen bonds (Table-2).

**Table 2:** Interaction, chemical bond, and binding energy between quercetin and integrins αvβ3, integrins α5β1, Caspase-3, Caspase-9, and VEGF.

Ligands	Protein	Binding energy (kJ/mol)	Point Interactions	Category	Type	Donor	Acceptor
Quercetin	αvβ3	−257,3	A: LYS409:HZ2 − :UNK0:O3	Hydrogen Bond	Conventional Hydrogen Bond	A: LYS409:HZ2	:UNK0:O3
			B: ARG261:HH11 - :UNK0:O7	Hydrogen Bond	Conventional Hydrogen Bond	B: ARG261:HH11	:UNK0:O7
			:UNK0:H29 − :UNK0:O4	Hydrogen Bond	Conventional Hydrogen Bond	:UNK0:H29	:UNK0:O4
			:UNK0:H31 − A: TYR224:O	Hydrogen Bond	Conventional Hydrogen Bond	:UNK0:H31	A: TYR224:O
			A: ARG99:CD − :UNK0:O4	Hydrogen Bond	Carbon Hydrogen Bond	A: ARG99:CD	:UNK0:O4
			A: SER160:CB − :UNK0:O6	Hydrogen Bond	Carbon Hydrogen Bond	A: SER160:CB	:UNK0:O6
Quercetin	α5β1	−226.1	A: THR258:HN − :UNK0:O2	Hydrogen Bond	Conventional Hydrogen Bond	A: THR258:HN	:UNK0:O2
			:UNK0:H28 − A: GLY255:O	Hydrogen Bond	Conventional Hydrogen Bond	:UNK0:H28	A: GLY255:O
			:UNK0:H29 - :UNK0:O4	Hydrogen Bond	Conventional Hydrogen Bond	:UNK0:H29	:UNK0:O4
			:UNK0:H31 − A: ASN256:OD1	Hydrogen Bond	Conventional Hydrogen Bond	:UNK0:H31	A: ASN256:OD1
			A: LEU257:HN − :UNK0	Hydrogen Bond	Pi-Donor Hydrogen Bond	A: LEU257:HN	:UNK0
			:UNK0 − A: LEU257	Hydrophobic	Pi-Alkyl	:UNK0	A: LEU257
			B: SER227:HG − :UNK0:H30	Unfavorable	Unfavorable Donor-Donor	B: SER227:HG	:UNK0:H30
Quercetin	Caspase 3	−262.9	A: THR270:HG1 − :UNK0:O5	Hydrogen Bond	Conventional Hydrogen Bond	A: THR270:HG1	:UNK0:O5
			B: ARG241:HE − :UNK0:O3	Hydrogen Bond	Conventional Hydrogen Bond	B: ARG241:HE	:UNK0:O3
			:UNK0:H29 − :UNK0:O4	Hydrogen Bond	Conventional Hydrogen Bond	:UNK0:H29	:UNK0:O4
			:UNK0:H32 − A: THR152:O	Hydrogen Bond	Conventional Hydrogen Bond	:UNK0:H32	A: THR152:O
			A: GLY153:CA − :UNK0:O1	Hydrogen Bond	Carbon Hydrogen Bond	A: GLY153:CA	:UNK0:O1
			A: LYS271:NZ − :UNK0	Electrostatic	Pi-Cation	A: LYS271:NZ	:UNK0
			A: LYS271:NZ − :UNK0	Electrostatic	Pi-Cation	A: LYS271:NZ	:UNK0
			A: GLY153:CA − :UNK0	Hydrophobic	Pi-Sigma	A: GLY153:CA	:UNK0
			:UNK0 − A: ILE187	Hydrophobic	Pi-Alkyl	:UNK0	A: ILE187
Quercetin	Caspase 9	−115.8	:UNK0:H29 − :UNK0:O4	Hydrogen Bond	Conventional Hydrogen Bond	:UNK0:H29	:UNK0:O4
			B: GLU187:CG − :UNK0:O5	Unfavorable	Unfavorable Bump	B: GLU187:CG	:UNK0:O5
Quercetin	VEGF	−213.4	:UNK0:H28 − A: GLN79:O	Hydrogen Bond	Conventional Hydrogen Bond	:UNK0:H28	A: GLN79:O
			:UNK0:H29 − :UNK0:O4	Hydrogen Bond	Conventional Hydrogen Bond	:UNK0:H29	:UNK0:O4
			A: PRO49:CD − :UNK0:O1	Hydrogen Bond	Carbon Hydrogen Bond	A: PRO49:CD	:UNK0:O1
			A: LYS48:CB − :UNK0	Hydrophobic	Pi-Sigma	A: LYS48:CB	:UNK0
			:UNK0 − A: PRO49	Hydrophobic	Pi-Alkyl	:UNK0	A: PRO49
			:UNK0 − A: PRO49	Hydrophobic	Pi-Alkyl	:UNK0	A: PRO49
			:UNK0 − A: PRO49	Hydrophobic	Pi-Alkyl	:UNK0	A: PRO49
			A: GLN79:O − :UNK0:C17	Unfavorable	Unfavorable Bump	A: GLN79:O	:UNK0:C17

### Chemical interactions occurred between quercetin and caspase-3/caspase-9

**Figure-3:**
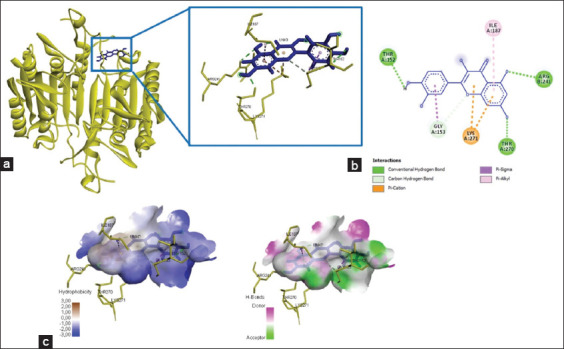
The three-dimensional and two-dimensional structure of the interaction between flavonoid compounds (quercetin) and caspase-3. (a) Three-dimensional structural interaction between flavonoid-ligand compound quercetin (blue) and caspase-3 on the active binding site (yellow). (b) This interaction is built by hydrogen and carbon-hydrogen bonds on the active site of caspase-3, namely, Thr270, Arg241, Thr152, Gly153, Lys271, and Ile187, which can be seen in the two-dimensional (b) and three-dimensional (c) structures.

**Figure-4:**
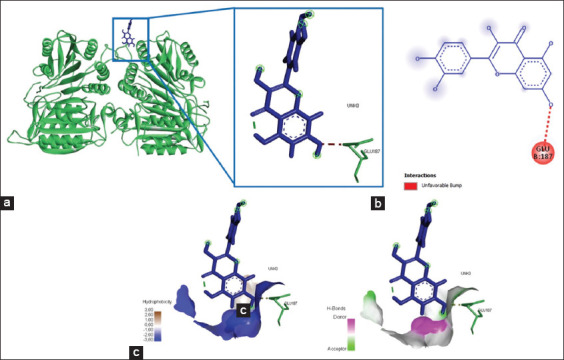
The three-dimensional and two-dimensional structure of the interaction between flavonoid compounds (quercetin) and caspase-9. (a) Three-dimensional structural interaction between flavonoid-ligand compound quercetin (blue) and caspase-9 on the active binding site (green). (b) This interaction is built by hydrogen and carbon-hydrogen bonds on the active site of caspase-9, namely, Glu187, which is seen in the two-dimensional (b) and three-dimensional (c) structures.

The interaction of caspase-3 with quercetin showed six amino acid residues bind to the amino acid residues of quercetin, namely, Thr270, Arg241, Thr152, Gly153, Lys271, and Ile187, with total energy formed of −262.9 kJ/mol (Figure-3 and Table-2). In addition, quercetin bound to caspase-9 at the amino acid residue Glu187 and yielded energy of −115.8 kJ/mol (Figure-4 and Table-2). The interaction between quercetin and caspase-3 and caspase-9 was formed by hydrogen bonds and Pi-cation, P-sigma, and Pi-alkyl interactions (Table-2).

### Quercetin binds to the active site of VEGF

**Figure-5:**
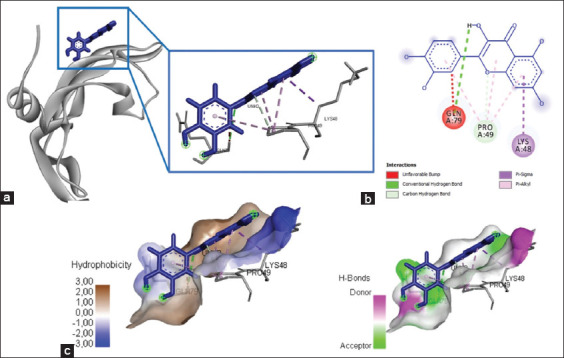
The three-dimensional and two-dimensional structure of the interaction between flavonoid compounds (quercetin) and vascular endothelial growth factor (VEGF). (a) Three-dimensional structural interaction between flavonoid-ligand compound quercetin (blue) and VEGF on the active binding site (gray). (b) This interaction is built by hydrogen and carbon-hydrogen bonds on the active sites of VEGF, namely, Gln79, Pro49, and Lys48, which can be seen in the two-dimensional (b) and three-dimensional (c) structures.

Quercetin interacted with the amino acid residues in VEGF, namely, Gln79, Pro49, and Lys48, and produced energy of −213.4 kJ/mol (Figure-5 and Table-2). Hydrogen bonds and Pi-cation, P-sigma, and Pi-alkyl interactions were the foundation of the chemical interactions between quercetin and VEGF (Table-2).

### Eugenol binds to the active sites of integrins αvβ3 and α5β1

**Figure-6:**
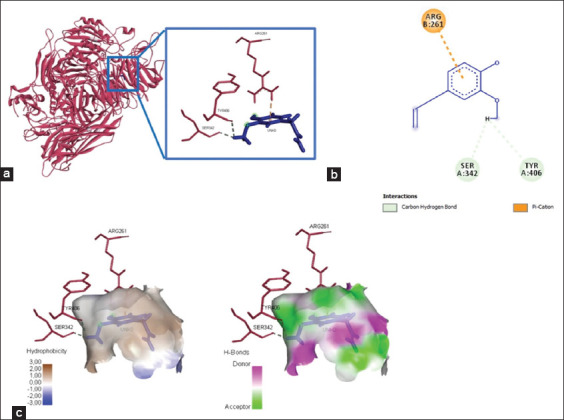
The visualization of the three-dimensional and two-dimensional interaction between phenolic compounds (eugenol) and integrin αvβ3. (a) Three-dimensional structural interaction between phenol-ligand eugenol compounds (blue) and integrin αvβ3 (red). (b) This interaction is established by hydrophobic hydrogen bonds (green) and aromatic bonds (yellow) on the active site of the vb3 integrin, namely, Ser342, Tyr406, and Arg261, which can be seen in the two-dimensional (b) and three-dimensional (c) structures.

**Figure-7:**
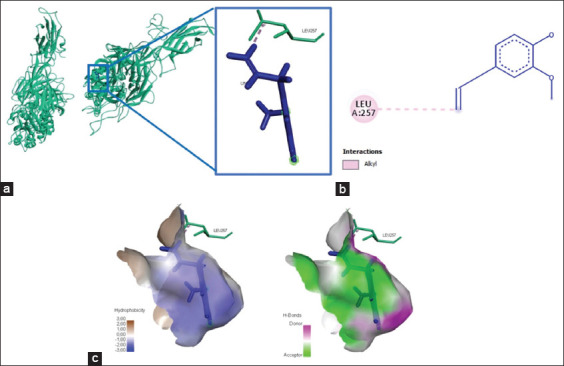
The visualization of the three-dimensional and two-dimensional interaction between phenolic compounds (eugenol) and integrin α5β1. (a) Three-dimensional structural interaction between phenol-ligand eugenol (blue) and integrin α5β1 (green). (b) This interaction is built by alkyl interactions (pink) on the active site of the α5β1 integrin, namely, Leu257, which is seen in the two-dimensional (b) three-dimensional (c) structures.

Three amino acid residues were visualized from the interaction between eugenol and the αvβ3 integrin, namely, Ser342, Tyr406, and Arg261; these bonds produced affinity energy of 181.6 kJ/mol (Figure-6 and Table-3). Meanwhile, the active compound in eugenol only bound to α5β1’s amino acid residue, Leu257, yielded energy of −169.2 kJ/mol (Figure-7 and Table-3). Hydrogen bonds and Pi-cation interactions established the chemical interactions between eugenol and integrins αvβ3 and α5β1 (Table-3).

**Table 3:** Interaction, chemical bond, and binding energy between eugenol and integrins αvβ3, integrins α5β1, caspase-3, caspase-9, and VEGF.

Ligands	Protein	Binding energy (kJ/mol)	Point Interactions	Category	Type	Donor	Acceptor
Eugenol	αvβ3	−181.6	:UNK0:H21 − A: SER342:O	Hydrogen Bond	Carbon Hydrogen Bond	:UNK0:H21	A: SER342:O
			:UNK0:H21 − A: TYR406:O	Hydrogen Bond	Carbon Hydrogen Bond	:UNK0:H21	A: TYR406:O
			B: ARG261:NH2 − :UNK0	Hydrogen Bond; Electrostatic	Pi-Cation; Pi-Donor Hydrogen Bond	B: ARG261:NH2	:UNK0
Eugenol	α5β1	−169.2	:UNK0:C12 − A: LEU257	Hydrophobic	Alkyl	:UNK0:C12	A: LEU257
Eugenol	Caspase 3	−184.6	B: ARG241:CD − :UNK0:O2	Hydrogen Bond	Carbon Hydrogen Bond	B: ARG241:CD	:UNK0:O2
Eugenol	Caspase 9	−90.9	:UNK0:H22 − C: GLU259:OE1	Hydrogen Bond	Conventional Hydrogen Bond	:UNK0:H22	C: GLU259:OE1
			:UNK0 − C: LEU240D	Hydrophobic	Pi-Alkyl	:UNK0	C: LEU240D
Eugenol	VEGF	−162.6	:UNK0 − A: LEU97	Hydrophobic	Pi-Alkyl	:UNK0	A: LEU97
			A: GLU38:CB − :UNK0:H19	Unfavorable	Unfavorable Bump	A: GLU38:CB	:UNK0:H19
			A: ASN75:HD22 − :UNK0:H22	Unfavorable	Unfavorable Donor-Donor	A: ASN75:HD22	:UNK0:H22

### Chemical interactions occurred between eugenol and caspase-3/caspase-9

**Figure-8:**
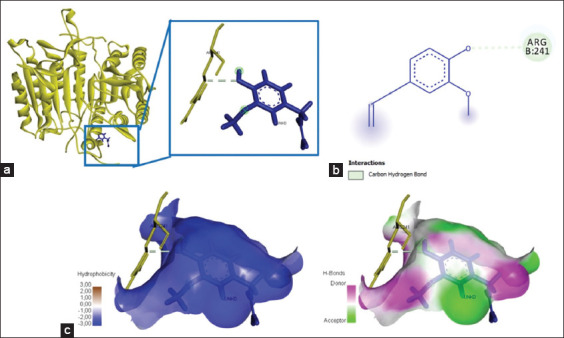
The visualization of the three-dimensional and two-dimensional interaction between phenolic compounds (eugenol) and caspase-3. (a) Three-dimensional structural interaction between the ligand eugenol (blue) and caspase-3 (yellow). (b) This interaction is established by hydrophobic hydrogen bonds on the active site of caspase-3, namely, Arg241, which is seen in the two-dimensional (b) and three-dimensional (c) structures.

**Figure-9:**
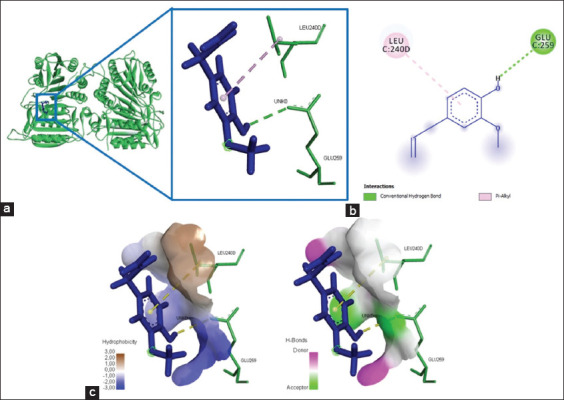
The visualization of the three-dimensional and two-dimensional interaction between phenolic compounds (eugenol) and caspase-9. (a) Three-dimensional structural interaction between the ligand eugenol (blue) and caspase-9 (green). (b) This interaction is established by hydrophobic hydrogen bonds on the active site of caspase-9, namely, Glu259 and Leu240, which can be seen in the two-dimensional (b) and three-dimensional (c) structures.

The interaction of the eugenol ligand and protein caspase-3 produced one amino acid residue (Arg241) that bound the eugenol ligand (Figure-8), yielded energy of −184.6 kJ/mol (Table-3). Furthermore, the binding of eugenol and caspase-9 involved two amino acid residues that bound to eugenol on the active site of caspase-9, namely, Glu259 and Leu240 (Figure-9). The interaction of eugenol and caspase-9 resulted in binding energy of approximately −90.9 kJ/mol (Table-3). The interaction between eugenol and caspase-3/caspase-9 was established through hydrogen bonds and Pi-alkyl interactions (Table-3).

### Eugenol bound to the active site of VEGF

**Figure-10:**
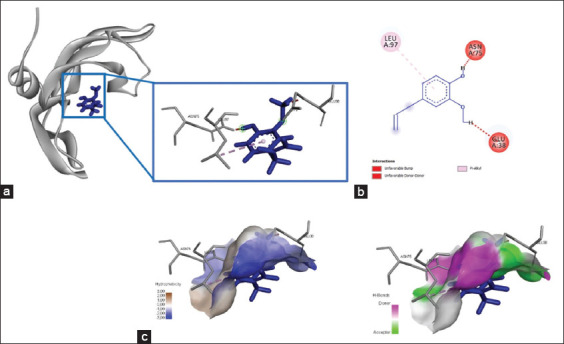
The visualization of the three-dimensional and two-dimensional interaction between phenol compounds (eugenol) and vascular endothelial growth factor (VEGF). (a) Three-dimensional structural interaction between the ligand eugenol (blue) and VEGF (gray). (b) This interaction is built by hydrophobic hydrogen bonds on the active site of VEGF, namely, Leu97, Glu38, Asn75, which can be seen in the two-dimensional (b) and three-dimensional (c) structures.

The interaction of the eugenol compounds and the VEGF protein indicated the involvement of three amino acid residues (Leu97, Glu38, and Asn75) that interacted with the eugenol compounds through Pi-alkyl and hydrophobic interactions (Figure-10 and Table-3). This interaction yielded a bond energy of −162 kJ/mol (Table-3).

### Decreasing the viability of A549 cells in the presence of EEOS

**Figure-11:**
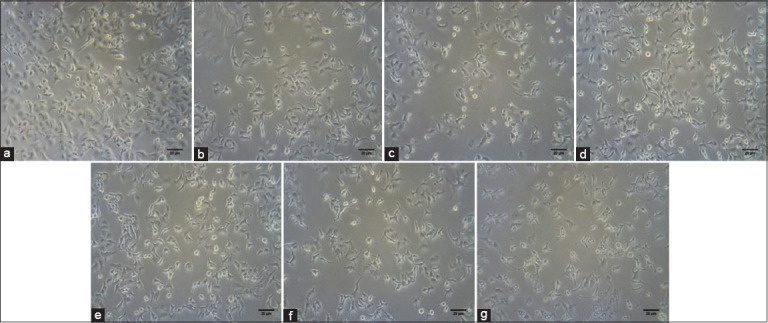
(a-g) A photomicrograph of the non-small cell lung cancer (NSCLC) (A549) cells’ viability. NSCLC (A549) cells were treated.

**Figure-12:**
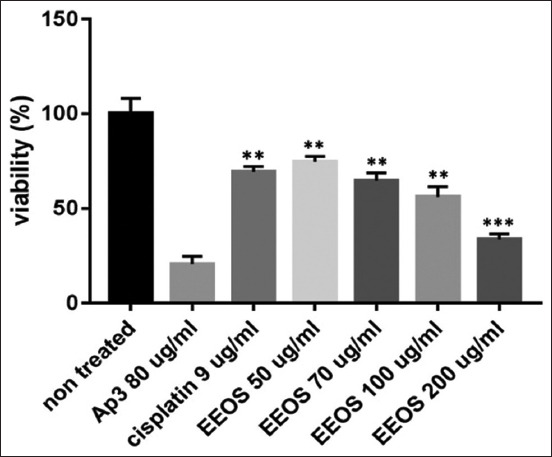
The inhibitory and antiproliferative effect of the ethanolic extract from Ocimum sanctum Linn. (EEOS) against non-small cell lung cancer (A549) cells. The cells were cultivated in the presence of an inhibitor (AP3) as the positive control, cisplatin as the commercial drug comparison, and EEOS at concentrations of 50, 70, 100, and 200 μg/mL. After 24 h, EEOS’s inhibitory effect was visualized by an MTT reagent at a wavelength of 595 nm (NT: Non-treated; ** significant p<0.0072; ***significant p<0.0002; n.s.=Not significant).

NSCLC cells were cultured to evaluate the ability of EEOS to inhibit the cells’ proliferation and adhesion to the extracellular matrix. Our results showed that EEOS significantly exhibited a cytotoxic effect in human A549 cells, demonstrated by the percent-mean viability decrement in a concentration-dependent manner similar to cisplatin, and compared with the untreated control. The optimal concentration of EEOS was 200 μg/mL; at this EEOS concentration, there was a smaller number of viable A549 cells than the number seen with other concentrations of EEOS (50, 70, and 100 μg/mL) (Figures-11 and 12).

## Discussion

Lung cancer remains the leading cause of cancer death in men and women worldwide [12]. In the past few years, the use of herbal medicine has continued to increase; currently, it is a trend to use herbal medicines concurrently with traditionally established treatments for cancer. One of the most popular herbs for medicinal uses is *O. sanctum* Linn.; however, not much has been known about its use in the treatment of lung cancer. In this current study, we used *in silico* molecular docking and *in vitro* approaches to determine the mechanism of action of the EEOS.

Spectrophotometry analysis showed that EEOS contained two primary compounds, that is, quercetin and eugenol, belonging to the flavonoid group and the phenol group, respectively (Table-1). Some research has shown that natural sources (eugenol, caffeic acid, gallic acid, apigenin, quercetin, and rosmarinic acid) have therapeutic benefits in treating various diseases, including cancer [13-17]. *In silico* molecular docking, analysis showed that quercetin and eugenol were able to bind to the active site of the αvβ3 integrin (Arg99, Arg261, Tyr224, Lys409, Ser342, Tyr406, and Arg261) and α5β1 integrin (Thr258, Gly255, Asn256, Leu257, Ser277, and Leu257). Integrins are known to play a crucial role in mediating the adhesion of epithelial cells to basement membranes. They also contribute to the migration, proliferation, and survival of tumor cells [18]. Furthermore, tumor cell expression of the integrins αvβ3, α5β1, a6b4, a4b1, and vb6 correlates with disease progression in various tumor types and is associated with cancer [18-20]. The adhesion of integrins to the extracellular matrix (ECM) provides the necessary traction for tumor cells’ invasion. Integrins contribute to tumor cells’ invasion by regulating the localization and activity of matrix-degrading proteases, such as matrix metalloprotease 2 (MMP2) and urokinase-type plasminogen activator (uPA). Integrin-mediated migration generally requires focal adhesion kinase and Src family kinase signaling [18,21,22]. Cancer treatment could be promoted by inhibiting the activity of the integrin complex [22,23]. In addition, integrins physically associate laterally with cell membrane proteins (e.g., CD151 or CD47) to elicit or modulate signaling [24,25]. Data from this study indicated the potential for inhibition by the ligand on the integrin αvβ3 and integrin α5β1 proteins. Inhibition of the integrin complex would impact the inhibition of the extracellular matrix adhesion (ECM) process and limit tumor cells’ invasion. In addition, inhibition of the process and activity of proteases and activators may occur. As a result, endothelial and epithelial cells rapidly undergo anoikis followed by inflammation and apoptosis when adhesion is disturbed [26,27]. The excessive growth of cancer cells is thus kept in check.

In addition, *in silico* molecular docking revealed the interaction between quercetin and eugenol with the active sites of VEGF, at positions Gln79, Pro49, Lys48, Leu97, Glu38, and Asn75. VEGF is a very potent pro-angiogenesis factor in the growth process of cancer cells that modulate the proliferation and migration of cancer cells [28]. In lung cancer, VEGF is overexpressed and plays an essential role in cancer cells’ growth and modulates other pro-angiogenic factors [12]. The binding between the active compounds in EEOS and the VEGF site through conventional hydrogen bonds is expected to inhibit the activity and stimulation. Our data are in line with the previous studies showing that the binding between VEGFB and VEGFR on the Pro143, Leu204, Phe172, Lys170, Pro173, Leu174, Lys171, Thr206, Glu208, Leu215, and Lys217 was able to inhibit interaction with the extracellular matrix. It is also known that *silico* molecular docking phytocompounds such as eriodyctiol, epicatechin, scutellarin, and ginkgolide A can bind to VEGF with tremendous energy as standard drugs for the 4T1 mammary carcinoma cell line [29,30]. We determined that the binding of eugenol and quercetin to VEGF resulted from the interaction of conventional hydrogen bonds and Pi-cation, Pi-sigma, and Pi-alkyl interactions, which significantly contributed to the stability of the bond structure [31-34]. Moreover, the binding interaction also supported the hydrogen’s donor and acceptor abilities and thus may predict the antioxidant activity of quercetin and eugenol [16,35].

Furthermore, if EEOS was able to inhibit αvβ3, α5β1, and VEGF, would EEOS also be able to induce apoptotic activity? Based on our molecular docking, it was found that quercetin and eugenol were able to interact with the active sites of caspase-3 and caspase-9 proteins, Thr270, Arg241, Thr152, Gly153, Lys271, and Ile187 and Glu187, Glu259, and Leu240, respectively. The key to EEOS’s pharmacological properties is its binding to the active site through conventional hydrogen bonds and Pi-cation, Pi-sigma, and Pi-alkyl interactions with amino acid residues in caspase-3 and caspase-9 proteins. Our *in silico* research found that quercetin and eugenol form stronger bonds in caspase-9 than caspase-3. Based on this binding, we postulated that EEOS could directly induce conformational changes in caspase-3 and caspase-9 to mature caspase-3 or cleaved caspase-3/caspase-9 to increase PARP’s cleavage activity, followed by anoikis, which, in turn, initiates apoptosis. Our results were consistent with the previous studies using natural ingredients, namely, chalcone 9X, *in silico*, to induce conformational changes in caspase-8 and caspase-3 to initiate apoptosis [35].

In accordance with the results of the *in silico* molecular docking, we performed an *in vitro* analysis. The *in vitro* results were consistent with the *in silico* data showing that the main contents of EEOS, namely, flavonoids and eugenol, were able to inhibit cells’ attachment to the extracellular matrix. EEOS may thus inhibit the adhesion of the NSCLC (A549) cells in the same way the conventional commercial drug cisplatin (used in this experiment) does. EEOS’s mechanism of action may inhibit adhesion, invasion, and cell migration, thus triggering anoikis and apoptosis in the A549 cell line.

## Conclusion

Our data revealed that EEOS could act as both an antiproliferative and apoptotic agent on NSCLC cells in *in silico* molecular docking and *in vitro* experiments. Nevertheless, further investigation of the mechanism, dosage, and other potential benefits of EEOS as a possible herbal medication to prevent NSCLC is needed.

## Authors’ Contributions

UK and TSDR: Experimental work and data analysis. NDAA: Literature study and wrote the manuscript. HW: Design of the experiment, literature study, wrote the concept of the original manuscript, and reviewing and editing of the final manuscript. DLK: Writing, reviewing, and editing of the manuscript. All authors read and approved the final manuscript.
